# Efficient removal of Pb(II) from aqueous solution by a novel ion imprinted magnetic biosorbent: Adsorption kinetics and mechanisms

**DOI:** 10.1371/journal.pone.0213377

**Published:** 2019-03-27

**Authors:** Yayuan He, Pian Wu, Wen Xiao, Guiyin Li, Jiecan Yi, Yafei He, Cuimei Chen, Ping Ding, Yanying Duan

**Affiliations:** 1 Xiang Ya School of Public Health, Central South University, Changsha,Hunan, China; 2 Hunan Institute of Food Quality Supervision Inspection and Research, Changsha, Hunan, China; 3 School of Life and Environmental Sciences, Guilin University of Electronic Technology, Guilin, Guangxi, China; 4 Xiangnan University, Chenzhou, Hunan, China; University of Eastern Finland, FINLAND

## Abstract

It is vital to understand the adsorption mechanisms and identify the adsorption kinetics when applying an adsorbent to remove heavy metals from aqueous solution. A Pb(II) imprinted magnetic biosorbent (Pb(II)-IMB) was developed for the removal of Pb^2+^ via lead ion imprinting technology and crosslinking reactions among chitosan (CTS), *Serratia marcescens* and Fe_3_O_4_. The effect of different parameters such as solution pH, adsorbent dosage, selectivity sorption and desorption were investigated on the absorption of lead ion by Pb(II)-IMB. The adsorbent was characterized by a Brunauer-Emmett Teller (BET) analysis, X-ray diffraction (XRD), vibrating sample magnetometry (VSM), scanning electron microscopy (SEM) and energy dispersive spectrometry (EDS). The adsorption kinetics, equilibrium and thermodynamics of Pb(II)-IMB for Pb(II) were studied. The results of the abovementioned analyses showed that the adsorption kinetic process fit well with the second-order equation. The adsorption isotherm process of Pb(II) on the Pb(II)-IMB was closely related to the Langmuir model. Thermodynamic studies suggested the spontaneous and endothermic nature of adsorption of Pb(II) by Pb(II)-IMB. The adsorption mechanism of Pb(II)-IMB was studied by Fourier transform infrared spectroscopy (FTIR) and X-ray photoelectron spectroscopy (XPS). The results indicated that the nitrogen in the amino group and the oxygen in the hydroxyl group of Pb(II)-IMB were coordination atoms.

## Introduction

Heavy metal pollution in the environment, which cause harmful effects on human health, has attracted much attention. Pb(II) constitutes the highest environmental hazard, especially in water, because of its widespread distribution, bioconcentration and physiological toxicity [1,2]. Pb(II) can induce kidney, liver and brain damage even at low concentrations [3–6]. Therefore, high efficient removal of Pb(II) ions could provide the basis for Pb(II) pollution prevention and protecting public health.

The traditional methods for removing Pb(II) include reduction [7], extraction [8], ion exchange [9], precipitation [10,11], and membrane separation [12], which suffer the problems of low efficiency and high operating costs. Adsorption is achieved by adsorbent combining with pollutants by physical and chemical attractive forces and is considered one of the most successful and economical technologies for removing contaminants from aqueous solution [13–15]. Various adsorbents, such as carbonaceous materials [16], minerals [17] and macromolecules [18], have been widely applied to remove Pb(II). Biobased adsorbents, as green adsorbents, are a promising material for treatment of Pb(II) contaminated water.

Chitosan (CTS), has caught the researchers’ attention because of its ecofriendliness, easy availiability and multifunctional chemical properties. Chitosan beads and chitosan-based composites have received increasing research attention for the purification of heavy metal contaminated water [19–23]. In addition, in the past few decades, the use of microorganisms as adsorbents for adsorbing and separating heavy metal ions has become a new research trend direction [24,25]. *Serratia marcescens* is a widely distributed microorganism in nature which can be cultured easily. They have been reported to possess the ability to adsorb organic matter [26,27] and toxic heavy metals [28–30] and are considered promising adsorbents. Based on the background detailed above, an absorbent prepared by combining chitosan with Serratia marcescens is a potential material for Pb(II) ion adsorption. The ability of this compound bio-adsorbent to remove heavy metals might be greatly improved compared with that of a single substance. However, this material which is similar to most biosorbents, presents the disadvantage of low separating efficiency, so it has limited its prospective practical use in removing pollutants [31,32].

In comparison with centrifugation, flocculation and filtration, magnetic separation technology without extra process provides a promising method for solid-liquid separation. As is known to us, Fe_3_O_4_ nanoparticles overcome the separation difficulty after use in terms of their superparamagnetic properties, which provides a new potential material for removing heavy metals from aqueous solutions with a low-cost but a high efficiency [33–36].

Moreover, the specificity and selectivity of the adsorbents will be greatly enhanced via the introduction of ion imprinting technology. The ion imprinting technique was developed based on the molecular imprinting technique [37–39]. Ion imprinting polymers (IIPs) for the removal or detection of heavy metals have been studied, and the results have demonstrated that the IIPs express strong affinity and excellent selectivity of the template ions [40–44].

This work aims to research the adsorption of Pb(II) by a Pb(II) imprinted magnetic biosorbent (Pb(II)-IMB). The effect of different parameters such as solution pH, adsorbent dosage, selectivity sorption and desorption were investigated on the absorption of lead ion by Pb(II)-IMB. The kinetic, equilibrium isotherms and thermodynamics were conducted to assess the removal performance of Pb(II) from water. The structure and adsorption mechanism of Pb(II)-IMB with Pb(II) was determined via BET, XRD, VSM, FTIR, and XPS. This information will be available for further applications in removing metal ions and other industrial technologies.

## Materials and methods

### Chemicals and reagents

The chemicals used in this experiment were provided by the Sinopharm Group Co., Ltd. (China) and of analytical grade. *Serratia marcescens* was purchased from the Shanghai Xinyu Biotechnology Co., Ltd. Magnetic nano-Fe_3_O_4_ were prepared in our laboratory.

### Synthesis of Pb(II)-IMB, nonimprinted magnetic biosorbent (NIMB) and imprinted magnetic absorbent (IMA)

Pb(II)-IMB and magnetic Fe_3_O_4_ were prepared according to previously reported methods [45]. CTS (0.19 g) was dissolved in 2% (w/w) acetic acid solution with mechanical shaking. 2 g/L Pb(NO_3_)_2_ (25 mL) were added into the solution. After stirring for 8 h at 25°C, the mixture was repeatedly washed with distilled water, ethanol and ether. Fe_3_O_4_ (0.43 g) and 0.4 mL epichlorohydrin were added and stirred for 3 h. One gram of bacteria powder, 2 wt% tripolyphosphate solution and 20 mL distilled water were added to the cross-linked solution. The Pb(II) imprinted in the material was eluted with 0.1 mol/L EDTA for 1 h in a vibrator. The mixture was immersed in 0.1 mol/L NaOH for 1 h and washed with distilled water until the pH of the washing water was neutral. Finally, Pb(II)-IMB was obtained after drying at 50°C in a vacuum oven.

The preparation of NIMB was similar to that of Pb(II)-IMB, except that lead-free solution was added during the synthesis procedure. Additionally, the preparation method of IMA can refer to the method of NIMB except that no bacteria powder was added during the preparation.

### Characterization

The specific surface area and total pore volume of the material were obtained using a standard Brunauer-Emmett Teller (BET) apparatus from Quantachrome Instruments company (model NOVA 1000e), and the pore size distribution was determined by the Barett-Joyner-Halenda (BJH) method. XRD patterns were recorded with an X-ray diffractometer using a Cu Kα spectral line at 40 kV and a 2θ range from 20 to 80^o^. The magnetic properties were analyzed by vibrating sample magnetometry (VSM). The surface morphology of Pb(II)-IMB was characterized by a scanning electron microscopy (SEM) coupled with an energy dispersive spectrometer (EDS). FTIR spectra were obtained to observe the complexation among the prepared materials using an Avatar-360 Fourier transform infrared spectrometer. DTGS KBr was used as the detector. XPS spectra were obtained by an X-ray photoelectron spectrometer (ESCALAB250Xi).

### Determination of point of zero charge (pH_pzc_)

Determination of pH_pzc_ was carried out according to the peported methods with some modifications [46]. 0.05 g Pb(II)-IMB and 50 mL of NaCl (0.01mol/L) solution were added in conical flasks, HCl (0.1 mol/L) was utilized to adjust the pH of the aqueous solution in the range of 3.0–7.0. then the mixed solution was shaken at a speed of 150 rpm at 25°C until the pH of solution doesn't change. According to the initial pH and the final pH value of differential (ΔpH) to judge the point of zero charge (ΔpH = 0).

### Batch experiments for Pb(II) removal

For Pb(II) adsorption studies, 0.05 g Pb(II)-IMB and 20 mL of Pb(II) solution were mixed in 50 mL conical flasks and shaken at a speed of 150 rpm in a thermostatic shaker. The experimental factors that may affect the adsorption efficiency were investigated, including the initial pH of the mixed solution (3.0~7.0), the biosorbents amount (1~ 25 g/L), the contact time (0~8 h), the initial concentration of Pb(II) (20~800 mg/L), and temperature (298~328 K). After adsorption, solid-liquid separation was performed using magnets, and the residual concentration of Pb(II) in the supernatant was detected by flame atomic absorption spectrometry (FAAS). The adsorption capacity and removal efficency of Pb(II)-IMB were counted according to *q*_*e*_ and *E*, respectively:

$$q_{e}=\frac{(C_{0}-C_{e})V}{m}$$ (1)

$$E=\frac{C_{0}-C_{e}}{C_{0}}\times 100\%$$ (2)

where *q*_*e*_ (mg/g) is the adsorption capacity of Pb(II), *C*_*0*_ and *C*_*e*_ (mg/ml) are the initial Pb(II) concentrations and equilibrium Pb(II) concentrations in aqueous solution, respectively. *V* (ml) is the volume of mixed solution, and *m* (g) is the weight of the Pb(II)-IMB added into the solution.

All experiments were repeated three times and the relative standard deviation (RSD) of experimental data was within 5%.

### Selectivity experiment

In order to investigate Pb(II) specificity of Pb(II)-IMB, Cu(II), Ni(II) and Cd(II) were selected as competitive ions. Pb(II)-IMB was mixed with the binary metal mixture solutions of Pb(II)/Cu(II), Pb(II)/Ni(II) and Pb(II)/Cd(II), and the concentration of each individual metal ion was 100 mg/L. The concentration of each individual metal ion was detected after adsorption equilibrium. The distribution ratio (D) and selectivity coefficient (K) were calculated as the followings:

$$D=\left(\frac{C_{0}-C_{e}}{C_{e}}\right)\frac{V}{m}$$ (3)

$$K=\frac{D\left(\mathrm{Pb}(\mathrm{II})\right)}{D\left(M(\mathrm{II})\right)}$$ (4)

where M(II) represents the competitive ion (Cu(II), Ni(II), Cd(II)).

## Results and discussion

### Point of zero charge (pH_pzc_) analysis

**Fig 1:**
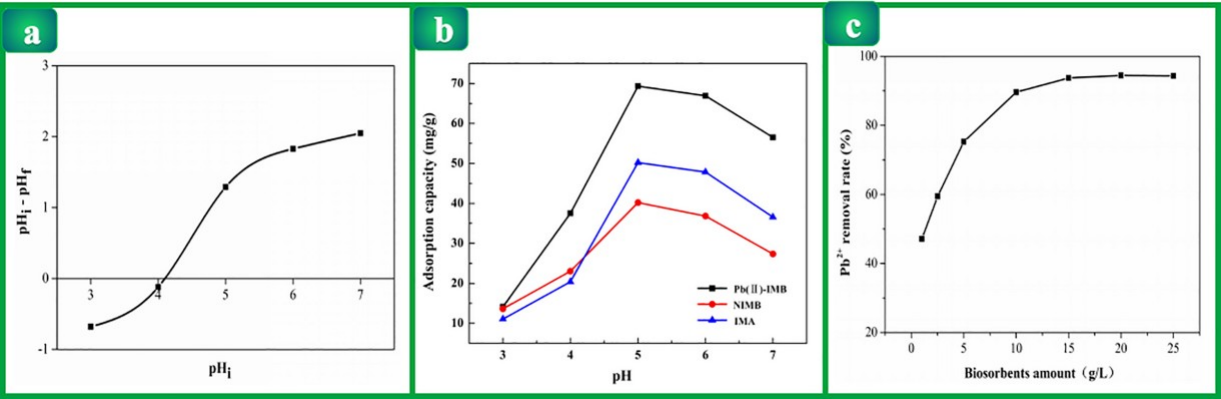
(a) pH_pzc_ of Pb(II)-IMB; (b) Curves of pH effect for Pb(II) adsorption on Pb(II)-IMB, NIMB and IMA; (c) The effect of adsorbent dosage on the adsorption of Pb^2+^ by Pb(II)-IMB.

The result of pH_pzc_ was shown in Fig 1A. It could be seen that the pH_pzc_ of Pb(II)-IMB was about 4.23. When the pH of the solution was lower than pH_pzc_, the surface of Pb(II)-IMB had a positive charge, which was conducive to the adsorption of anionic species. On the contrary, the negative charge of the Pb(II)-IMB would be favored at pH higher than the pH_pzc_. The optimal PH for Pb(II)-IMB was 5 which was higher than pH_pzc_. Thus, it could support the experimental result that the adsorption efficiency of Pb(II) was the maximum at the pH of 5.

### Effect of pH value on the adsorption process

The pH value in aqueous solution plays a crucial role in the adsorption of metal ions because of the competitive adsorption of hydrogen ions(H^+^) and metal ions. H^+^ can ionize the functional group, consequently, affecting the adsorption ability of the adsorbent to the metal ions. Therefore, the influence of Pb(II)-IMB on the adsorption capacity of Pb(II) was investigated in the pH range of 3.0–7.0. The experimental results are shown in Fig 1B. As the initial pH changed from 3 to 5, the adsorption ability of Pb(II)-IMB increased while the pH increased, and the maximum adsorption efficiency was achieved at pH = 5. When the initial pH value was higher than 5, the adsorption capacity gradually decreased. The chelation reactions among R-NH_2_ of Pb(II)-IMB, Pb(II) and H^+^ under different pH conditions are illustrated as follows:

$$\begin{array}{l} R-NH_{2}+\frac{1}{n}Pb^{2+}\Leftrightarrow R-NH_{2}\frac{1}{n}Pb^{2+}\\ R-NH_{2}+H^{+}\Leftrightarrow R-NH_{3}{}^{+} \end{array}$$

At low pH conditions, the concentration of H^+^ is relatively high, the functional groups (-NH_2_) of Pb(II)-IMB are protonated by H^+^ to form -NH_3_^+^, therefore the chelation reaction between Pb(II) and -NH_2_ will be carried out in the reverse direction. In addition, the formation of -NH_3_^+^ can cause electrostatic repulsion between the positively charged functional groups and the adsorbates [47], which greatly reduces the adsorption ability of Pb(II)-IMB for Pb(II). As the pH value increased, the concentration of H^+^ in the solution decreased, and the chelating effect between -NH_2_ and metal ions was dominant compared to the complexation between -NH_2_ and H^+^. When the pH of the solution was in the range of 4 to 5, the amino group of Pb(II)-IMB was present in the form of -NH_2_, which induced electrostatic attraction between Pb(II)-IMB and Pb(II). The adsorption capacity of Pb(II)-IMB increased. When the pH value was in the range of 5 to 7, the concentration of OH^-^ in the solution increased. The dissociation of the -OH on the surface of Pb(II)-IMB led to a negative charge on the surface of the particle. However, due to the presence of OH-, the Pb^2+^ in aqueous solution would changed to Pb(OH)_2_, which hindered the adsorption of Pb(II). When the initial pH value was higher than 5, the adsorption capacity gradually decreased.

The adsorption capacity of the nonimprinted magnetic biosorbent (NIMB) and imprinted magnetic absorbent (IMA) for Pb(II) are also shown in Fig 1B. Their adsorption behavior was similar to Pb(II)-IMB. However, the maximum adsorption capacity of Pb(II)-IMB to Pb(II) was significantly better than that of NIMB and IMA.

### Effect of adsorbent amount

The influence of adsorbent amount on Pb(II) adsorption is shown in Fig 1C. For a certain concentration of lead ion solution, the available adsorption sites increased with the increase of adsorbent dosage. Therefore, the removal rate improved. However, with the increase of adsorbent dosage from 15 g/L to 25 g/L, the increase in the removal rate tended to be flat. Because Pb(II) ions were adsorbed by a large amount of adsorbent, which lowered the equilibrium concentration of the solution, and reduced mass transfer between Pb(II)-IMB and lead ions. So, when adsorbent dosage was continuously increased, the adsorption sites were difficult to combine with lead ions, which resulted in little change of removal rate.

### Adsorption kinetic studies

**Fig 2:**
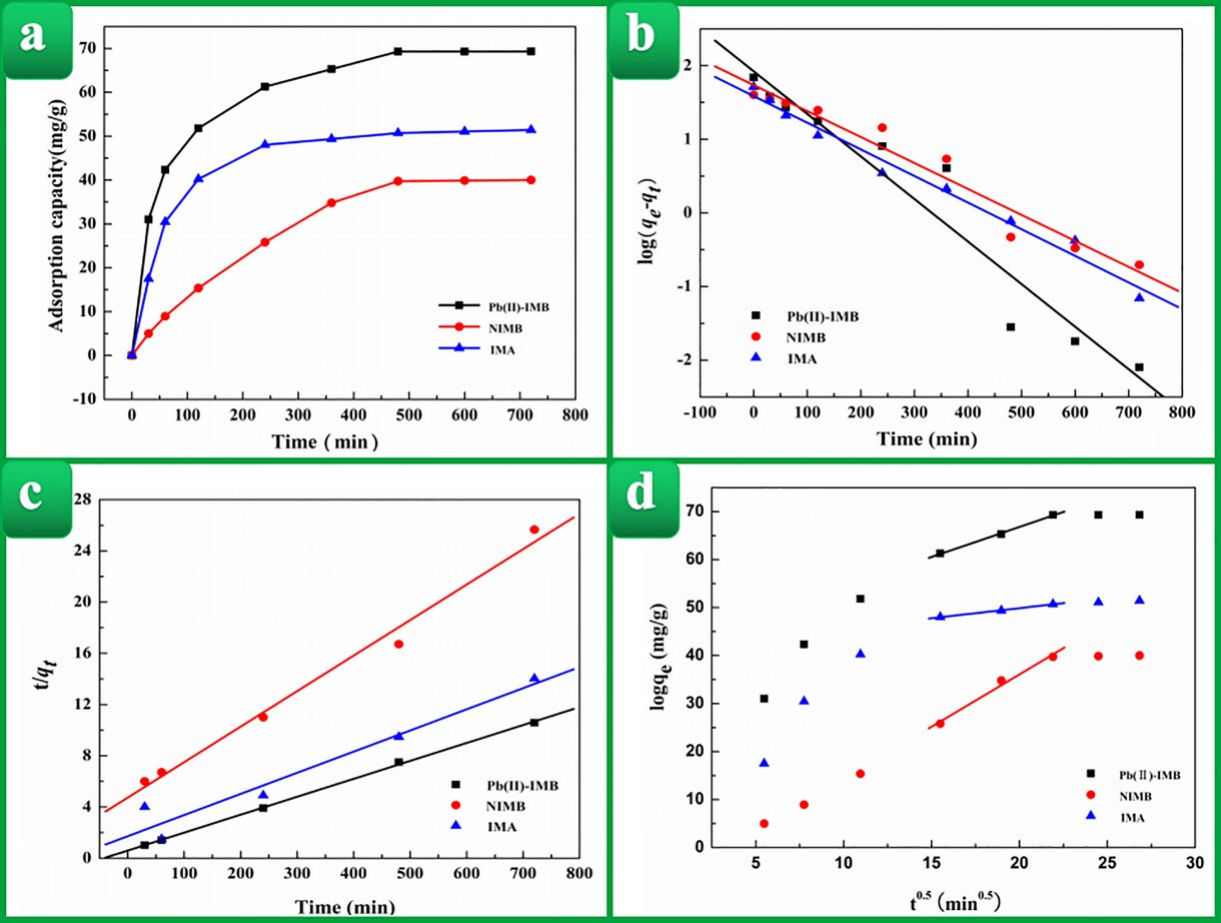
(a) The effect of the contact time on the adsorption of Pb(II) by Pb(II)-IMB, NIMB, IMA; (b) Pseudo-first-order curve of Pb(II) adsorption onto Pb(II)-IMB, NIMB, IMA; (c) Pseudo-second-order curve of Pb (II) adsorption onto Pb(II)-IMB, NIMB, IMA; (d) Intraparticle diffusion model curve of Hg Pb (II) adsorption.

The influence of reaction time on the removal ability of Pb(II)-IMB for Pb(II) (*C*_*0*_ = 200mg/L, pH = 5.0) is shown in Fig 2A. The adsorption capacity of Pb(II)-IMB increased rapidly in the first 120 min because more active sites for adsorption of Pb(II) could be obtained. In the following time, the adsorption capacity of Pb(II)-IMB increased slowly and reached the adsorption equilibrium state at 480 min, due to the occupation of the available adsorption sites. The influence of other biosorbents (NIMB and IMA) on adsorption performance was also examined so that the contributions of the ion imprinted technology and *Serratia marcescens* in Pb(II) removal could be assessed. As shown in Fig 2A, compared to NIMB and IMA, Pb(II)-IMB exhibited better adsorption performance. The adsorption capacity of the prepared biosorbent was noticeably improved after the incorporation of Pb^2+^ imprinting and *Serratia marcescens*.

Additionally, the pseudo-first-order, pseudo-second-order equations and the Webber’s pore-diffusion model were applied to explore the mechanisms of the adsorption processes [48,49]. The equation of the pseudo-first-order kinetic model is displayed in Eq (5) as follows:

$$\log(q_{e}-q_{t})=\log q_{e}-\frac{k_{1}}{2.303}t$$ (5)

where *q*_*e*_ and *q*_*t*_ (mg/g) are the adsorption capacity of Pb(II) on the adsorbent at equilibrium and at time t (min), respectively. *k*_*1*_ (min^-1^) is the rate constant of first-order adsorption. The linear plot of log (q_e_-q_t_) versus t was utilized to determine the rate constant k_1_ and the correlation coefficient R^2^ of Pb(II) at different concentration ranges.

The equation of the pseudo-second-order kinetic model was presented in Eq (6) as follows:

$$\frac{t}{q_{t}}=\frac{1}{k_{2}q_{e}{}^{2}}+\frac{t}{q_{e}}$$ (6)

where *q*_*e*_ and *q*_*t*_ (mg/g) represent the count of the adsorbed Pb(II) on the biosorbent at equilibrium and at time t (min), respectively. *k*_*2*_ (g/mg.min) is the rate constant of second-order adsorption. The straight-line plots of t/q_t_ against t could be drawn according to the kinetic experimental data if the adsorption process conforms to the quasi-second-order adsorption kinetic model.

The Webber’s pore-diffusion model originated from Fick’s second law of diffusion can be described as:

$$q_{t}=k_{i}t^{0.5}$$ (7)

where *k*_*i*_ is the Webber’s pore-diffusion model constant (mg/(g.min)). The *k*_*i*_ is the slope of straight-line portions of the plot of *q*_*t*_ against *t*^*0*.*5*^.

The fitting curves of log (*q*_*e*_*-q*_*t*_) and t/q_t_ versus t are shown in Fig 2B and 2C, respectively. The relevant kinetic model parameters for Pb(II) adsorption are displayed in Table 1. The results showed that the correlation coefficient (R^2^) of the pseudo-second-order kinetic equation was high, indicating that the experiment closely obeyed to the second-order model. Hence, Pb(II)-IMB adsorption of Pb(II) was a chemical adsorption, and adsorption rate was controlled by adsorption sites. Additionally, the results also showed that Pb(II)-IMB possessed the fastest adsorption rate of Pb(II) among the three adsorbents, suggesting that the ion imprinting technique and *Serratia marcescens* could not only improve the adsorption capacity but also enhance the adsorption rate of the adsorbent.

**Table 1:** Kinetic parameters of Pb (II) removal onto Pb (II)-IMB, NIMB, IMA.

Adsorbents	q_e_ (mg/g)	Pseudo-first-order kinetic parameters			Pseudo-second-order kinetic parameters		
		q_cal_ (mg/g)	K_1_(×10-2min^-1^)	R^2^	q_cal_ (mg/g)	K_2_(×10-4g/mg.min)	R^2^
Pb(II)-IMB	69.34	69.337	1.336	0.9334	70.082	3.324	0.9968
NIMB	40.21	40.178	0.806	0.9543	42.484	1.140	0.9739
IMA	51.50	51.530	0.829	0.9826	51.980	4.230	0.9989

It was notable that the pseudo-second-order kinetic model derived from chemical reaction were not considering the adsorbates diffusion process. Therefore, the Webber’s pore-diffusion model was carried out. The fitting curves qt versus *t*^*0*.*5*^ were shown in Fig 2D, it could be find that the intercept values calculated from the fitting results were clearly not zero, so particle diffusion was not the solely step to control the adsorption process, which agreed well with the kinetic analysis. The internal diffusion rate constant *k*_*i*_ of Pb(II) adsorption by three adsorbents was the largest with NIMB (2.1825mg/(g.min)), followed by Pb(II)-IMB (1.2501mg/(g.min)), and finally IMA (0.4202mg/(g.min)).

### Adsorption isotherm studies

**Fig 3:**
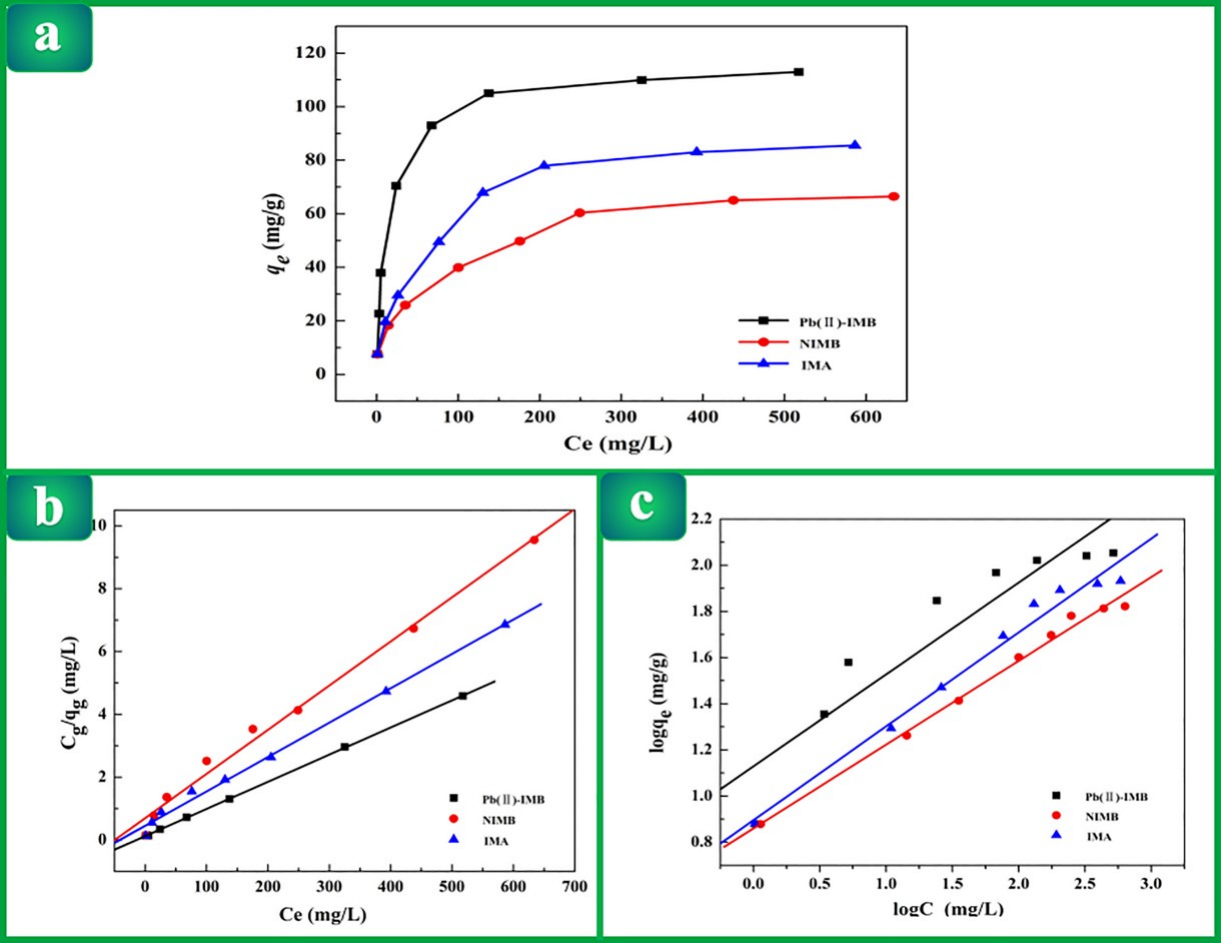
(a) Adsorption isotherm of Pb(II) adsorption onto Pb(II)-IMB, NIMB, IMA; (b) Linear plot of Langmuir isotherm; (c) Linear plot of Freundlich isotherm.

The adsorption isotherms of Pb(II)-IMB, INMB and IMA are shown in Fig 3A. The adsorption ability distinctly increased with increasing initial Pb(II) concentrations. The adsorption isotherms also illustrated that the maximum adsorption capacity of Pb(II)-IMB is significantly higher than that of INMB and IMA. Using adsorption isotherms to express the reciprocal action between adsorbent and solute could offer crucial information for optimizing the practical use of Pb(II)-IMB. Thus, two of the most commonly used isothermal adsorption models were selected in our study.

The Langmuir isothermal adsorption model assumed that the adsorbent was a monomolecular layer with a uniform surface, and there was no interaction between the adsorbates. The linear expression of the Langmuir adsorption isotherm model is exhibited in Eq (8) as follows:

$$\frac{C_{e}}{q_{e}}=\frac{C_{e}}{q_{m}}+\frac{1}{K_{L}q_{m}}$$ (8)

where *q*_*e*_ (mg/g) is the amount of Pb(II) adsorbed by adsorbents when the adsorption reached equilibrium, *q*_*m*_ (mg/g) represents the saturated adsorption capacity of the adsorbents per unit mass, *C*_*e*_ is the concentration of remaining adsorbate in the solution, and *K*_*L*_ (L/mg) is the Langmuir constant.

Alternatively, the Freundlich isothermal adsorption model, with no limitation on the monomolecular layer or uniform surface of adsorbents, was also used to model the experimental isotherm data. The log-linearized Freundlich equation is shown in Eq (9) as follows:

$$\log q_{e}=\log K_{F}+\frac{1}{n}\log C_{e}$$ (9)

where *q*_*e*_ and *C*_*e*_ are the same as the definition in Eq (8), n is a constant depicting the adsorption intensity, and K_F_ is the Freundlich constant representing the adsorption capacity.

The linear regression of the Langmuir adsorption isothermal model and the Freundlich adsorption isothermal model of Pb(II) adsorption onto Pb(II)-IMB, NIMB and IMA are shown in Fig 3B and 3C, respectively. The parameters and coefficient of correlation (R^2^) for the Langmuir and Freundlich models are displayed in Table 2. It can be observed that the Langmuir equation best described the adsorption isotherms, indicating that the adsorption process was monolayer adsorption. The reason may be that the active sites are relatively evenly distributed on the surface and inside of the adsorbents.

**Table 2:** Langmuir isotherm and Freundlich isotherm parameters of Pb(II) removal onto Pb(II)-IMB, NIMB, IMA.

Adsorbents	Langmuir isotherm model			Freundlich isotherm model		
	q_m_(mg/g)	K_L_	R^2^	K_F_	n	R^2^
Pb(II)-IMB	116.279	0.0691	0.9999	13.452	2.512	0.8662
NIMB	70.922	0.0201	0.9905	7.236	2.754	0.9913
IMA	91.743	0.0241	0.9946	7.861	2.459	0.9798

Based on the Langmuir isotherm model, the maximum adsorption capacity of Pb(II)-IMB was 116.279 mg/g for Pb(II). Compared with other absorbents already reported in the literature (Table 3), Pb(II)-IMB showed a larger adsorption capacity for Pb(II).

**Table 3:** Comparison of adsorption capacity of Pb (II)-IMB for Pb(II) sorption with other adsorbents reported in the literature.

Adsorbent	Adsorption capacity (mg/g)	Ref.
Xanthate-modified cross-linked magnetic chitosan/poly(vinyl alcohol) particles (XCMCP)	59.86	[50]
ungrafted Pb(II) ion-imprinted polymers (RAFT-IIP)	53.8	[51]
Magnetic modified vermiculite	70.4	[52]
Fe_3_O_4_@DAPF core-shell ferromagnetic nanorods (CSFMNRs)	83.3	[53]
Pb(Ⅱ)-IMB	116.279	This study

### Effect of temperature

Adsorption thermodynamics is used to study the effect of temperature on adsorption. The thermodynamic parameters include Gibbs free energy (ΔG), entropy change (ΔS) and enthalpy change (ΔH).

$$K_{d}=\frac{q_{e}}{C_{e}}$$ (10)

$$lnK_{d}=\frac{\Delta S}{R}-\frac{\Delta H}{RT}$$ (11)

$$\Delta G=-RT\ln K_{d}$$ (12)

Where *K*_*d*_ is the thermodynamic equilibrium constant, *R* is the ideal gas constant (8.314 J/mol K), *T* is the thermodynamic temperature (*K*). Table 4 lists the thermodynamic parameter values.

**Table 4:** Thermodynamic parameters for Pb(II) adsorption on Pb (II)-IMB.

Temperature(K)	ΔG(KJ/mol)	ΔH(KJ/mol)	ΔS(J/mol K)
298	-190.77	11.91	0.68
308	-197.55		
318	-204.34		
328	-211.15		

From the Table 4, negative ΔG indicated that Pb(II) adsorption was spontaneous, and its value decreased with the increase of temperature. Positive ΔH value indicated that the adsorption was an endothermic reaction, and increasing the temperature could facilitate the adsorption. The positive ΔS showed that the degrees of freedom increased at the solid-liquid interface during the adsorption and it might be involved with the substitution of water hyderation molecules of Pb(II) by functional groups.

### Competition adsorption

**Fig 4:**
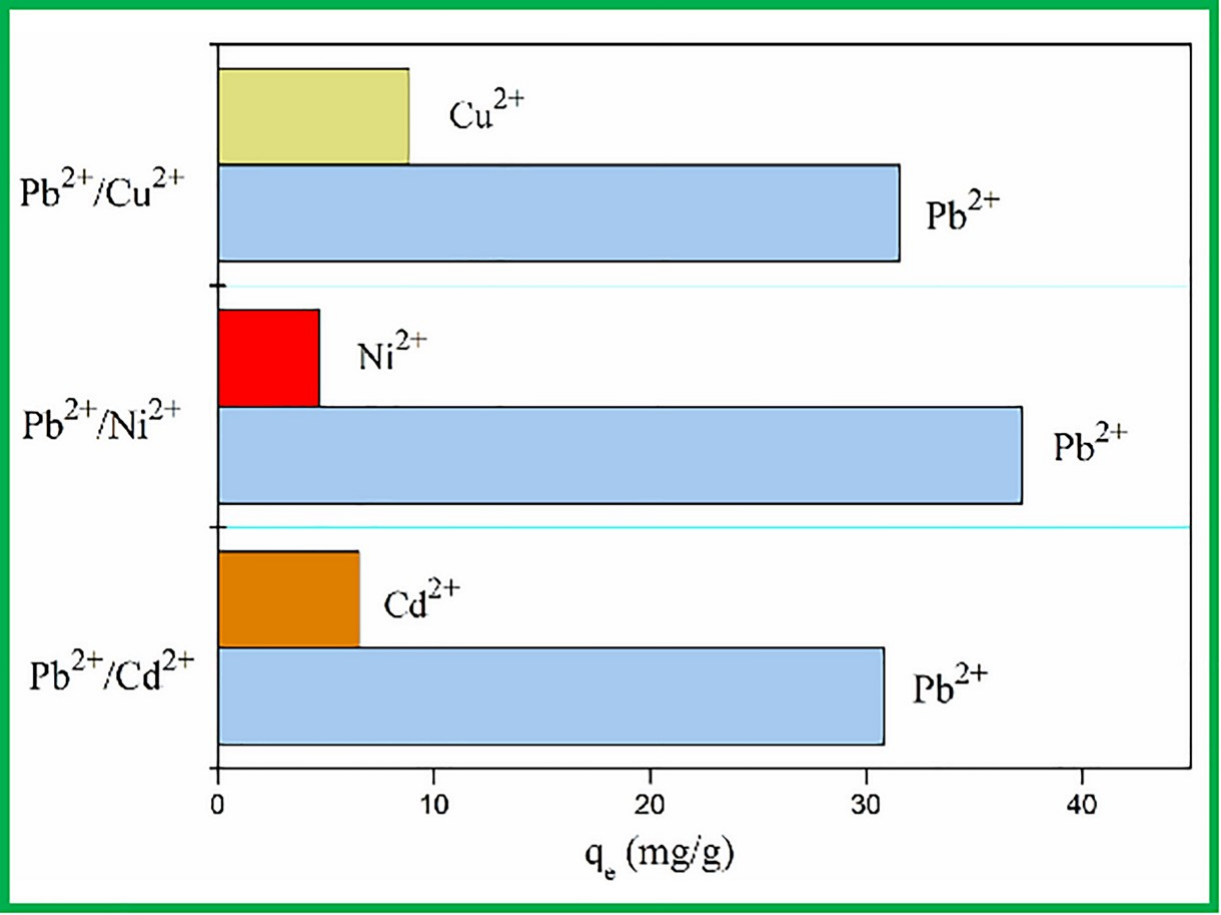
Comparative adsorption of competitive ions onto the Pb(II)-IMB.

Cu^2+^, N^i2+^, and Cd^2+^ were selected as competitive ions because they had the same number of charges and similar ionic radii. The competitive adsorption of Pb(II)-IMB from their binary metal mixture solutions of Pb(II)/Cu(II), Pb(II)/Ni(II) and Pb(II)/Cd(II) were performed and the results are shown in Fig 4. It was clear that the adsorption capacity of Pb(II) was well above that of the other metal ions. Moreover, the distribution radio of Pb(II)-IMB was much larger than that of the other metal ions. The selectivity coefficient of Pb(II)-IMB for Pb(II)/Cu(II), Pb(II)/Ni(II) and Pb(II)/Cd(II) are 3.56, 7.94 and 4.71, respectively. The results indicated that Pb(II)-IMB had strong affinity to Pb(II) ion from aqueous solution containing other competitive ions.

### Desorption Pb(II) ion and reusability of Pb(II)-IMB

It is vital to study the desorption of Pb(II) ions from the material due to the fact that the adsorbed Pb(II) ions can not only be separated from Pb(II)-IMB, but the material can also be regenerated so that it can continue to adsorb lead ions. Pb(II)-IMB was repeatedly adsorbed-desorbed five times, using 0.1 mol/L of EDTA as eluent and NaOH as regenerant. After five adsorption-desorption cycles, the adsorption capacity of the reused Pb(II)-IMB did not decrease significantly, and the recycled material was about 15% loss at the 5th cycle. The results indicated that Pb(II)-IMB had reusability and stability for Pb(II) adsorption.

### Characterization of Pb(II)-IMB

#### Surface area and pore size analysis

**Fig 5:**
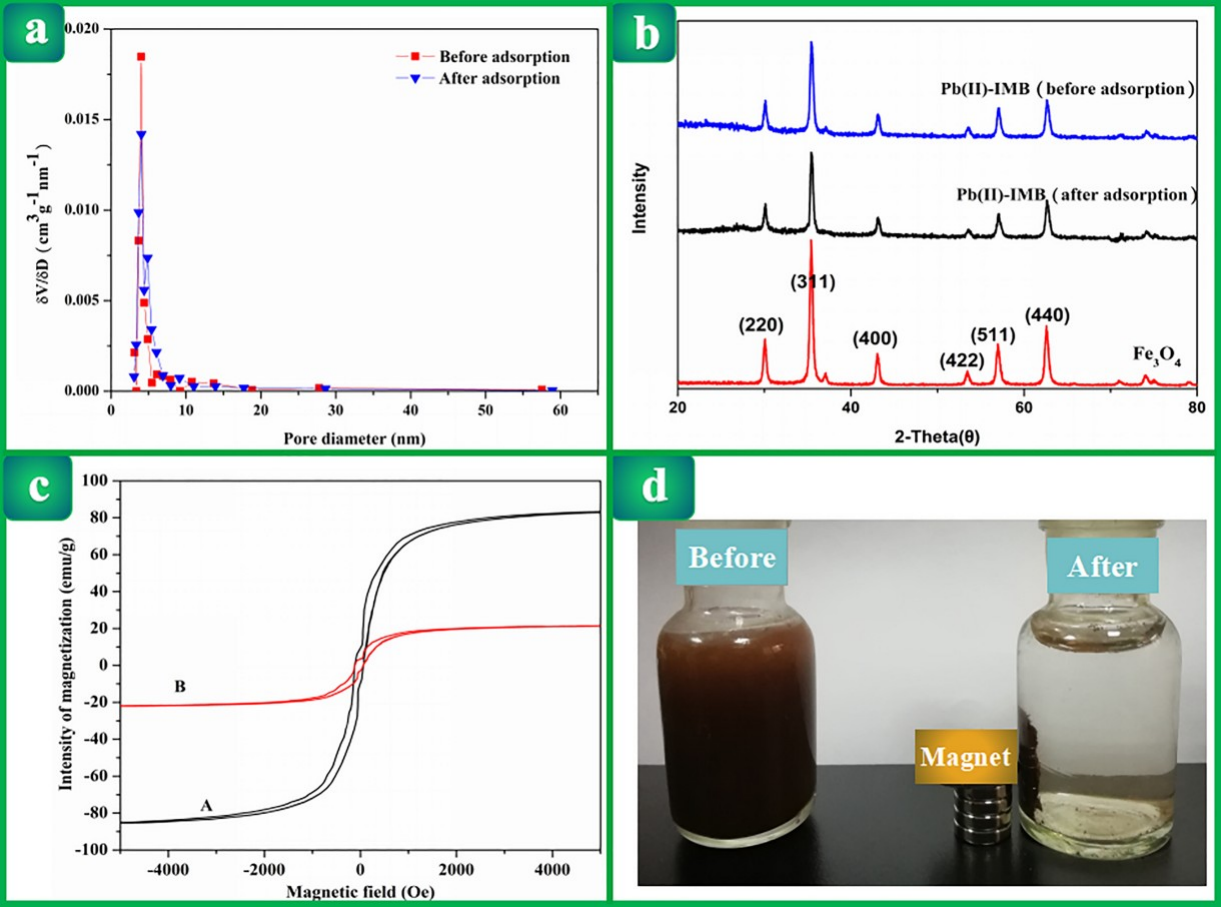
(a) Pore size distribution (δV/δD) for Pb(II)-IMB before and after adsorption of Pb(II); (b) XRD spectra of Pb(II)-IMB before and after Pb(II) adsorption and XRD spectrum of Fe_3_O_4_; (c) The magnetic hysteresis loop of Fe_3_O_4_ (A) and Pb(II)-IMB (B); (d) Pb(II)-IMB suspension before and after an external magnetic field was applied.

Compared with Pb(II)-IMB (16.813 m^2^/g), the material adsorption of lead ions showed a larger specific surface area (20.083 m^2^/g) after adsorption of lead ions, demonstrating that the specific surface area changed, which could be due to the fact that Pb(II) ions were combined with active sites on the absorbent. The average pore size of Pb(II)-IMB was approximately 4.1 nm, and the pore volume was 0.0239 cc/g. The pore size distributions (Fig 5A) indicated that the diameters of the pores in the material were between 3.4 and 8.7 nm, revealing that the material was a mesoporous material. The pore size before and after adsorption changed little.

#### XRD analysis of Pb(II)-IMB

XRD can be used to express the crystallinity of the material. From the Fig 5B, the diffraction peaks of the material before and after adsorption are identical. Pb(II)-IMB showed a crystalline structure with several sharp peaks ((220), (311), (400), (422), (511) and (440), respectively) and was generally similar to that of Fe_3_O_4_. These results demonstrated that the crystal structure of the material mainly came from Fe_3_O_4_, and the incorporation of Fe_3_O_4_ during the preparation of the material does not interfere with the magnetism of Fe_3_O_4_ and has no effect on the structure of Fe_3_O_4_. The diffraction peaks of Pb(II)-IMB (2θ = 30.06°, 35.42°, 42.99°, 53.48°, 57.04° and 62.62°) were weaker than those of Fe_3_O_4_, suggesting that intramolecular hydrogen bonding of amino and hydroxyl groups of CTS in Pb(II)-IMB weakens the degree of crystallization of the material.

#### Magnetism analysis Pb(II)-IMB and Fe_3_O_4_

The magnetization curves of Fe_3_O_4_ and Pb(II)-IMB were measured at 300 K with a VSM. As shown in Fig 5C. The reversible coercivity and remanence of the hysteresis loop were close to zero, indicating that both Fe_3_O_4_ and Pb(II)-IMB have superparamagnetism. The saturation magnetization (M_s_) of Pb(II)-IMB was 21.5 emu/g, which was lower than that of the bare Fe_3_O_4_ (M_s_ = 83.25 emu/g). This resulted from the fact that Fe_3_O_4_ only accounts for a portion of Pb(II)-IMB. However, the saturation magnetization of Pb(II)-IMB was sufficient for it to be recovered under an applied magnetic field (Fig 5D).

#### SEM and EDS analysis of Pb(II)-IMB

**Fig 6:**
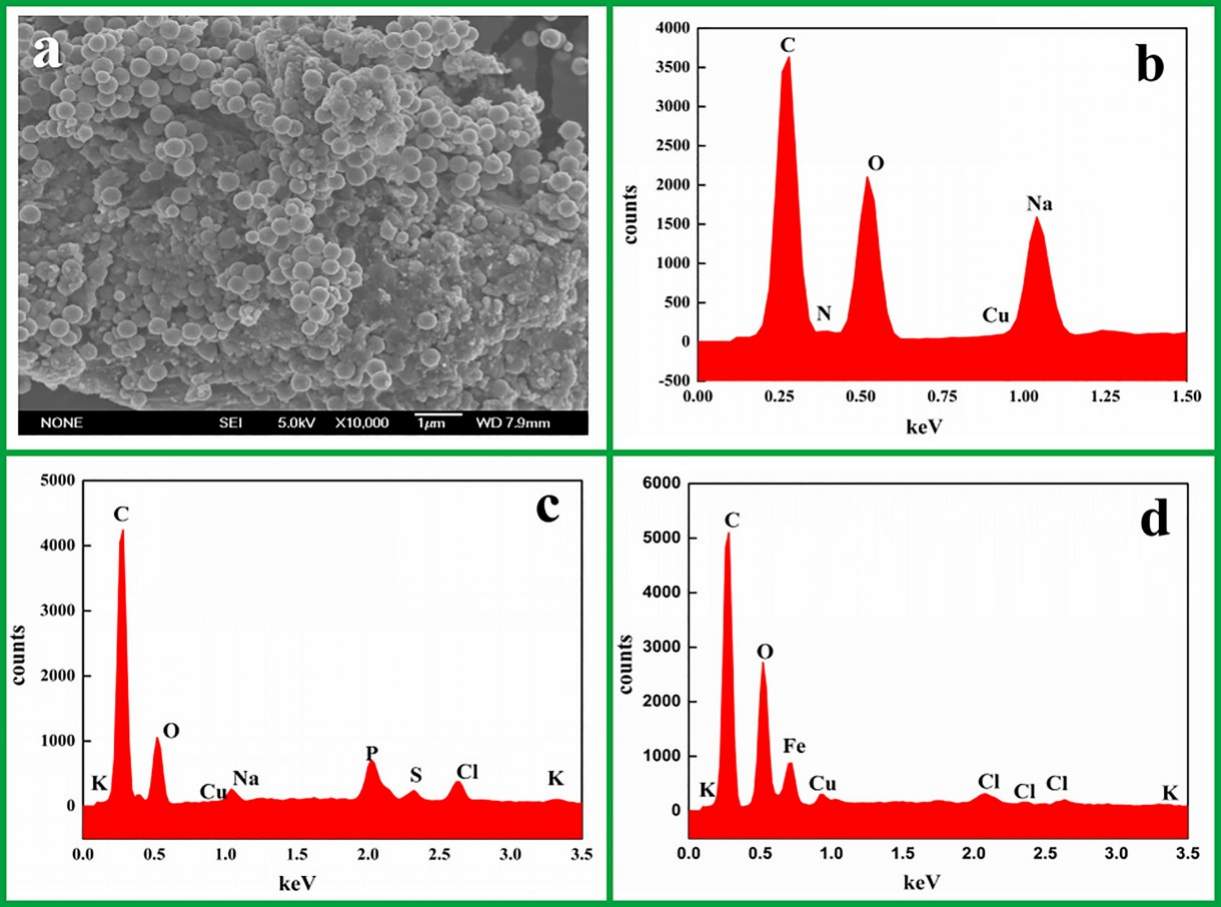
(a) SEM of Pb(II)-IMB before Pb(II) adsorption (×10000); (b-d) EDS results of chitosan, *Serratia marcescens* and Pb(II)-IMB.

The SEM and EDS results of Pb(II)-IMB are shown in Fig 6. As shown in Fig 6A, the surface of Pb(II)-IMB was uneven, which was favorable for the adsorption of Pb(II). The EDS patterns of chitosan, *Serratia marcescens* and Pb(II)-IMB are displayed in Fig 6B–6D, respectively. The EDS spectra of CTS showed a dominant presence of C, O and Na, while *Serratia marcescens* contained extra P, S, Cl, K. The peaks of C, O, Na, Fe, Cl, K were all detected in Pb(II)-IMB, indicating that chitosan, *Serratia marcescens* were successfully introduced into Pb(II)-IMB.

#### FTIR spectral analysis of Pb(II)-IMB

**Fig 7:**
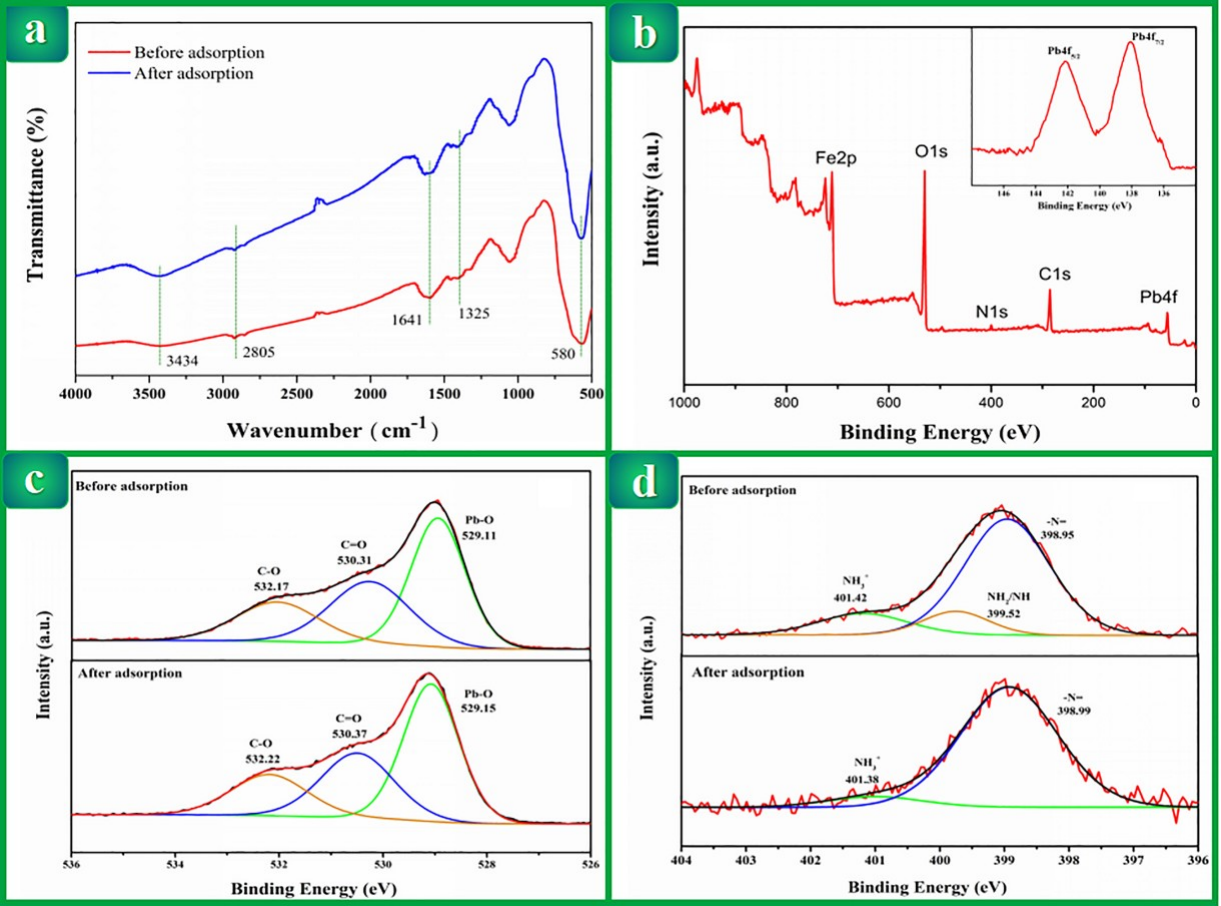
(a) FT-IR spectra for Pb(II)-IMB before and after adsorption of Pb(II); (b) XPS spectra of Pb(II)-IMB after adsorption; O 1s (c) and N 1s (d) narrow XPS scan for Pb(II)-IMB before and after adsorption.

The FTIR spectra before and after adsorption were generally similar, but several characteristic peaks had shifted. Fig 7A shows the FTIR spectra of the material before and after adsorption of lead ions. Broad peaks could be observed in the spectra at approximately 3434 cm^−1^ (-OH stretching and -NH_2_ stretching) and shifted to a lower frequency, indicating that the hydroxyl or amino groups participated in complexation. The peaks at approximately 2805 cm^-1^ (C-H stretching of -CH_3_ and -CH_2_) and near 1641 cm^-1^ (C = O stretching) shifted, which indicated that the crosslinking had reacted. The increase of the absorption peak at 1325 cm^-1^ was regarded as a characteristic peak of the coalescence of CTS and Pb(II), illustrating that the acetamide groups interacted with Pb(II). The peaks observed at 580 cm^-1^ confirmed the presence of Fe-O stretching, indicating that the nano-Fe_3_O_4_ were successfully embedded in the materials. Meanwhile, FTIR analysis suggested that the structure of Pb(II)-IMB remained relatively intact after Pb(II) adsorption.

#### XPS analyses of Pb(II)-IMB before and after adsorption

The XPS binding energies acquired from the C 1s, O 1s, N 1s, Pb 4f of materials are listed in Table 5. The binding energies of C 1s, N 1s, O 1s were likely to change because of the crosslinking reaction involving -NH_2_ and -OH and the introduction of mycelium. As seen in Table 5, the XPS spectra of C 1s did not obviously indicate notable changes in binding energy (BE) before and after Pb adsorption. Neither FTIR nor XPS spectra provided distinct evidence of a significant change in the chemical bond linked to the carbon atom after Pb adsorption. It was possible that the effect of Pb-carbon interaction on the Pb adsorption of the materials was mostly due to nonspecific interactions or feeble chemical interactions.

**Table 5:** The binding energy of C 1s, O 1s, N 1s, Fe 2p and Pb 4f in Pb(II)-IMB.

Pb(II)-IMB	C 1s	O 1s	N 1s	Fe 2p	Pb 4f
Before adsorption	284.91	529.66	399.25	710.53	
After adsorption	284.99	530.87	399.01	710.73	138.69

Fig 7B shows the XPS wide scan spectrum of Pb(II)-IMB after Pb adsorption. In combination with the change of the binding energy of Pb in Table 5, the change of BE provides evidence that lead is successfully adsorbed on the surface of material.

The O1s spectrum of Pb(II)-IMB was resolved into three single peaks at 529.1 eV, 530.3 eV, and 532.2 eV, corresponding to Pb-O,–C = O, and C–O (Fig 7C), demonstrating that the oxygen atom of the carbonyl group and hydroxamic acid in Pb(II)-IMB coordinated with Pb. In addition, the binding energy of O1s in Pb(II)-IMB was shifted from 529.66 eV to 530.87 eV. The generated positive shift of 1.21 eV provided evidence of the interaction between O and Pb in adsorption: electrons were further donated to Pb, which made the O atoms electron-deficient.

The N1s XPS spectra of Pb(II)-IMB before and after Pb(II) adsorption are displayed in Fig 7D. Obviously, the first predominant peak, presented at approximately 398.9 eV, is characteristic of the–N = , which proves that the cross-linking agent does connect CTS to the mycelium by reacting with amino groups on the surface of the CTS and mycelium. The second one, with a lower intensity, is located at higher binding energies, approximately 401.4 eV, and corresponds to NH_3_^+^. There is a peak of approximately 399.52 eV (-NH_2_/NH) in the image before adsorption, which disappears after adsorption. The possible reason is that the acylation reaction occurred during the adsorption process.

### Possible simulation structure of Pb(II)-IMB

**Fig 8:**
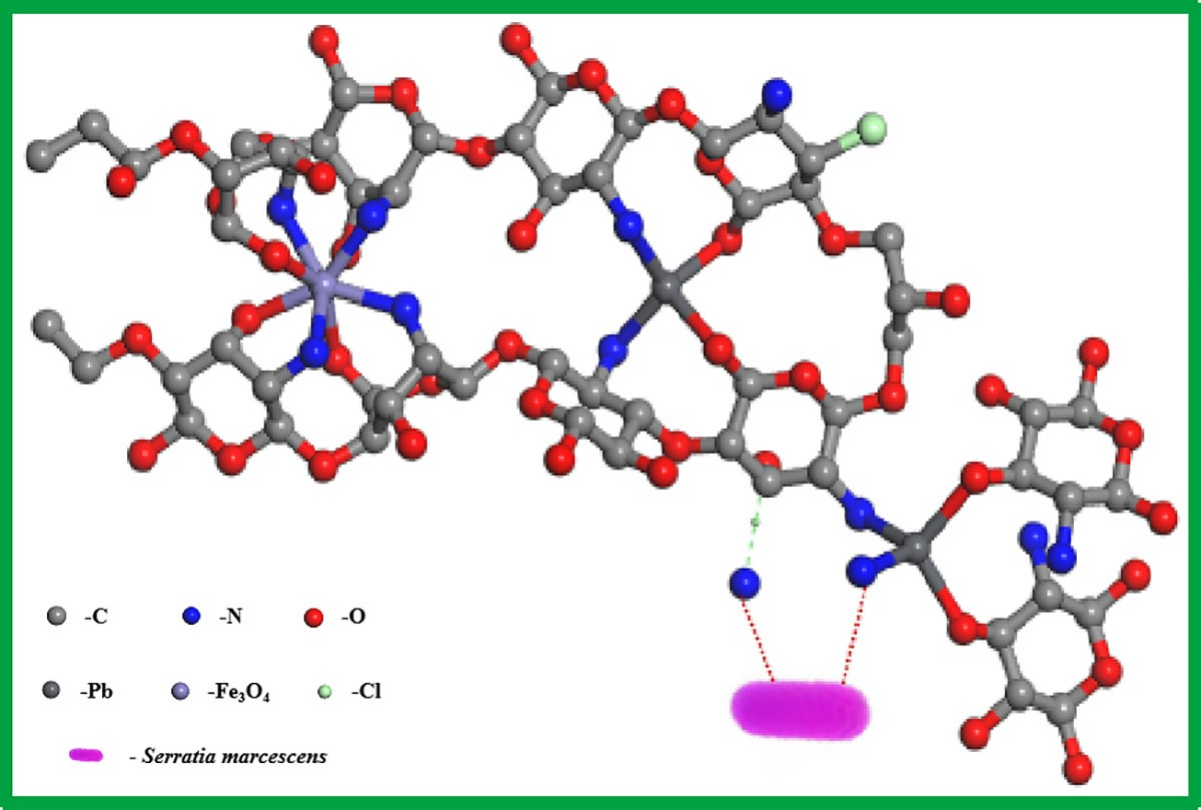
Possible structure of Pb(II)-IMB after adsorption.

According to the above characterization analysis, the molecular structure of the adsorbed material can be inferred (Fig 8). From the kinetics and material mechanisms, it could be speculated that the adsorption of Pb(II) by Pb(II)-IMB was efficient.

## Conclusion

In this work, characteristics of pH effect, adsorbent dosage, competitive ion adsorption and regeneration for Pb(II)-IMB were tested. The results showed that Pb(II)-IMB was very effective in removing Pb(II) ions from aqueous solution at pH 5, and Pb(II)-IMB had good selectivity for Pb(II) ions. Moreover, the adsorbent could be used many times without significant reduction in adsorption capacity. Adsorption kinetics, equilibrium studies and and thermodynamics were applied to research the adsorption behavior of Pb(II)-IMB for Pb(II). The experimental results showed that the adsorption kinetic curve matched well with the second-order equation, suggesting that the adsorption rate was controlled by chemical adsorption sites. The adsorption isotherm model was closely related to the Langmuir model. The adsorption process was a spontaneous endothermic process. BET, XRD, VSM, SEM, EDS, FTIR and XPS were utilized to explore the structure and adsorption mechanisms of Pb(II)-IMB. The results showed that Pb(II) formed a chelated structure with nitrogen in the amino group and oxygen in the hydroxyl group of Pb(II)-IMB, which could explain the adsorption behavior of Pb(II) on the material. In conclusion, Pb(II)-IMB exhibits a variety of good performances such as efficient adsorption capacity, good selectivity, excellent reproducibility and convenient separation under magnetic field. So, Pb(II)-IMB can be considered as promising candidate for selective adsorption of Pb(II) ions from wastewater.
